# Remediating Garden Soils: EDTA-Soil Washing and Safe Vegetable Production in Raised Bed Gardens

**DOI:** 10.3390/toxics10110652

**Published:** 2022-10-29

**Authors:** Christoph Noller, Wolfgang Friesl-Hanl, Rebecca Hood-Nowotny, Andrea Watzinger

**Affiliations:** Department of Forest- and Soil Sciences, Institute of Soil Research, University of Natural Resources & Life Science (BOKU), 1180 Vienna, Austria

**Keywords:** heavy metals, soil washing, remediation, recultivation, biochar, vermicompost

## Abstract

Soil remediation is an important practice in the restoration of heavy metal-contaminated soils and reduce the heavy metal exposure of the local population. Here, we investigated the effect of an ex-situ soil washing technique, based on ethylenediaminetetraacetic acid (EDTA) as a chelating agent, on a contaminated Cambisol. Lead, Cd and Zn were investigated in different soil fractions, drainage water and four vegetables from August 2019 to March 2021. Three treatments consisting of (C) contaminated soil, (W) washed soil and (WA) washed soil amended with vermicompost and biochar were investigated in an outdoor raised bed set up. Our results showed that the total and bioavailable metal fractions were significantly reduced but failed to meet Austrian national guideline values. Initial concentrations in the soil leachate increased significantly, especially for Cd. Vegetables grown on the remediated soil took up significantly lower amounts of all heavy metals and were further reduced by the organic amendment, attaining acceptable values within EU guideline values for food safety. Only spinach exceeded the thresholds in all soil treatments. The increase in soil pH and nutrient availability led to significantly higher vegetable yields.

## 1. Introduction

Interest in urban agriculture has grown among practitioners and scientists, in part due to its social and economic benefits [1], but specifically due to growing urban populations, loss of arable land, malnourishment and climate change [2,3]. Whilst the contribution of urban agriculture to improved food security in highly populated areas is still discussed [4,5], the physical, mental and public health aspects are widely acknowledged [6,7]. For this reason, the promotion of urban agriculture is part of a bigger strategy of the European Commission with the view towards a growing ‘green infrastructure’ [8]. Community lots and green strips in the inner-urban perimeter are often favored by garden projects due to their central location [9] but are often exposed to previous industrial use or have proximity to roadways; such spaces are likely to contain elevated concentrations of heavy metals (HM) [10,11]. On a worldwide scale, the main source of HMs are active or abandoned mines or the associated heavy industry [12]. Private gardens found in their vicinity are impacted directly by mining activities [13], but can also be located along rivers polluted by mining industry [14,15].

If exposed to HM emission, soils end up as a direct sink for these pollutants since they are not decomposable and they accumulate, with constant inputs from atmospheric deposition especially in the fine fraction (<2 µm) [16]. These fractions can be transported over great distances and cause biotoxicity [17]. High levels of Pb exposure in early childhood are still occurring [18] and even the uptake of low concentrations can cause neurological and behavioral deficits especially in children [19]. While the ingestion and inhalation of re-suspended dust from contaminated soil is among the main pathways of human exposure to HM, the intake via home-grown vegetables from contaminated soil poses an additional thread to human health [20,21]. Therefore, the remediation of contaminated garden soils has the potential to increase small scale vegetable production and improve public health.

There is a great variety of remediation technologies to treat contaminated sites, primarily soil excavation, metal immobilization and soil washing. Immobilization approaches are generally effective and low-cost but long-term immobility has to be monitored constantly to prevent future environmental risks [22]. In practice, traditional dig and dump approaches as well as physical separation are still widely used, and treated soils are lost or strongly degraded in the process, while soil-preserving technologies such as soil washing still lack relevance [23]. Chemical soil washing has already been discussed as an possible solution in the early 1990s [24]. Its impact on the soil pedological properties is less invasive compared to traditional washing approaches due the desorption of HM from the clay fraction, which can be preserved [15,25]. However, washing also has adverse effects on the bioavailability of HM and can decrease soil fertility and microbial activity [26,27,28,29]. To mitigate these secondary effects, (in-)organic amendments are common post-treatments [30,31]. A wide variety of extraction agents including chelating agents, surfactants, inorganic agents and organic acids [25,32] have been investigated in wide concentration ranges [33,34] over the last decades, aiming to increase metal extraction and rehabilitation of the treated soil; however, no chemical soil washing technique has been applied on a realistic scale yet.

One technology that applies ethylenediaminetetraacetic acid (EDTA) to extract HMs from contaminated soil is ReSoil^®^. Its technological aspects on metal removal and washing solution recycling have already been demonstrated in multiple studies [23,35]. Different chelating agents, various post-treatments [29,36] and their impact on physical and biological properties were included [28,37]. Biodegradable chelators were studied as an alternative to EDTA [38] but have been shown to be ineffective due to their fast degradation during the washing process, reducing their efficiency and increasing material costs [36,39]. The growth of vegetables on remediated soil has been investigated in pot experiments [40,41] and despite the progress in soil remediation efficiency and promising results from plant experiments, the application of EDTA soil washing techniques is still manly conceptual [32,42]. To understand how upscaling the washing process as well as its application in the field will impact the efficiency of HM removal, bioavailability, and ground water leaching, field experiments need to be conducted to study this technology under natural conditions. Two experiments were set up to study soils highly contaminated with Pb, Cd and Zn, washed using ReSoil^®^ in a raised bed garden set up to close this knowledge gap. The first pilot scale experiment on calcareous soils has been conducted in Prevalje, Slovenia and was published in 2021 [43,44,45]. The study presented here is the second published garden scale application and investigated an acidic soil. When compared to calcareous soils, the amount of EDTA can be cut by half (from 120 to 60 mmol kg^−1^) to reach similar heavy metal extraction, however, the changes in soil chemistry for acidic soils are much stronger with respect to soil pH, due to the formation of gypsum during soil washing. This makes it difficult to extrapolate the results to residual EDTA and hence to HM dynamics.

In this paper, we explore the effects of soil washing and amendments on a private garden scale. We study the change in soil physiochemical properties but also the HM plant uptake and resulting yields of different vegetables and cultivars over a longer time frame and under realistic conditions. Three soil amendments have been applied after the washing treatment to stabilize residual EDTA, rehabilitate the deteriorated soil structure and revitalize the soil microbial community [37]. (1) The immobilization of residual EDTA after zero valent iron (ZVI) amendment was investigated over the study period. While the use of ZVI has become obligatory in ReSoil^®^ to curb residual EDTA–metal complexes in the solid soil fraction [29], it has not been studied over a longer time period. (2) To rehabilitate the soil microbial life, vermicompost has been used as an inoculant for degraded soil [46], while also introducing nutrients removed by the remediation. (3) Biochar was applied due to its favorable effects on soil physical properties, especially in soil with a poor structure [47]. To determine the potential EDTA escape into deeper soil layers, we improved an existing colorimetric method and measured concentrations in soil water as well as leachate over the study period and discussed the interaction of EDTA and HM mobility and plant uptake.

## 2. Materials and Methods

### 2.1. Soil

The soil used in this experiment originated from Arnoldstein, a former smelting region in Austria, and is heavily contaminated with Pb, Cd and Zn. The contamination is well known among the local population; nevertheless, vegetable production in private gardens is still common practice [13,48]. The soil was collected from pastureland (46°33′15.8″ N 13°40′58.4″ E) and after removing the top grass layer, the upper 20 cm of soil was excavated (ca. 12 t) and transported to the soil washing facility.

The EDTA soil washing was conducted at the Envit Ltd. pilot scale remediation facility in Prevalje, Slovenia, which utilized the ReSoil^®^ soil treatment technology. It was patented by Domen Lestan [49] and is described in detail by Gluhar et al. (2021) [43]. Briefly, stones (>25 cm) were separated from the soil and added back after the treatment. The contaminated soil was washed in 1000 kg batches with 1000 L of Na–EDTA solution (60 mmol kg^−1^). Sand and gravel (>5 mm) were removed by wet sieving and washed separately. The fine fraction (<5 mm) was amended with 10 kg of ZVI before being transferred to the filter press. To remove mobile chelates and HM, the soil was rinsed with fresh water inside the press. The soil blocks formed by hydraulic pressure were homogenized using an agricultural rotator and the stones were reintroduced. The washing solution was recycled by addition of CaO (pH > 12) that substituted the heavy metals bound to ETDA by Ca. Paper waste was added to the solution to adsorb the heavy metals and was subsequently removed by filtration. To recover the EDTA for the next washing batch, H_2_SO_4_ was added to bring the solution back to pH 2, resulting in the formation of CaSO_4_.

### 2.2. Preliminary Cultivar Selection Experiment

To test for differences in HM plant uptake between the washed and untreated soils, vegetable species and varieties were selected: two leafy vegetables (*Spinacia oleracea* L. and *Lactuca sativa* L.) and two root vegetables (*Raphanus raphanistrum* subsp. *sativus* L. and *Daucus carota* L. subsp. *sativus* (Hoffm.) Arcang.). For each species, common varieties were selected (spinach: 13, lettuce: 12, radish: 13, carrots: 9, Figures S4–S7).

In these initial experiments, plants were grown in 550 mL pots (*n* = 4) under controlled greenhouse conditions with a 14 h light period and approximate temperatures of 25 °C for daytime and 20 °C for nighttime. Radish, carrots, and lettuce were grown in the contaminated soil and irrigated daily to maintain moisture levels close to field capacity. Spinach was grown in a hydroponic experiment using perlite and a nutrient solution spiked with Pb as PbNO_3_ (0.331 mg L^−1^) and Cd as CdSO_4_ (0.208 mg L^−1^). Seedlings were thinned to three plants after germination. Radish, lettuce and spinach were grown for 5 weeks while carrots were grown for 13 weeks. At harvest, the above and below ground plant biomass was separated, washed, weighed, and dried at 60 °C until a constant weight. The dry material was ground using a stainless steel ball-mill, acid digested and analyzed for HM. Cultivars were selected for the raised bed experiment according to the lowest HM uptake.

### 2.3. Raised Bed Experiment

**Figure 1:**
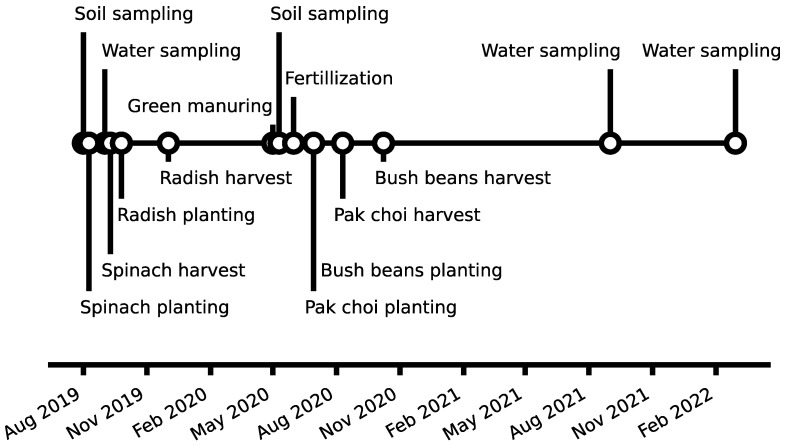
Timeline and events of the raised bed experiment.

The vegetable garden experiment was set up in 2019 and comprised twelve raised beds (Figure S1) with dimensions of 1 × 2 × 0.5 m (L × W × H). A 0.5 mm PVC lining prevented drainage water penetrating the natural soil. Two separate drainage tubes with a sampling point at both ends of the beds were installed at the bottom. The ground level of the beds was constructed to form a water-divide in the center. The seepage layer consisted of 5 cm washed quartz gravel (4–8 mm), covered by 2 cm quartz sand (0.3–2 mm) and was filled up with 40 cm soil (approximately 1 t) in August 2019 (Figure 1). To prevent uncontrolled precipitation, a transparent roof was installed, and the beds were equipped with a drip irrigation system, SMT100 soil water content sensors (Truebner GmbH, Neustadt, Germany) and jet-fill tensiometers (Soilmoisture Equipment Corp., Goleta, CA, USA) to control irrigation for optimal plant growth. In March 2020, each bed was divided into two subplots. In one half, Phacelia (*Phacelia tanacetifolia* Bentham) was grown as a soil improvement measure. After two months, the plants were harvested, homogenized, and reintroduced as green manure. After vegetables on the contaminated control treatment showed dwarfish growth, all beds were fertilized with 100 kg N ha^−1^, 40 kg P_2_O_5_ ha^−1^ and 160 kg K_2_O ha^−1^ in late May 2020 to account for possible nutrient deficiencies.

Three treatments were investigated using quadruplicates (*n* = 4): the original contaminated soil (C), the EDTA-washed soil (W) and a washed variant (WA) amended with vermicompost (VC) and biochar (BC). W and WA were homogenized in a trommel sieve (Figure S2). The VC was produced from food waste and horse manure and acquired from a local farmer and analyzed by Eurofins Lebensmittelanalytik Österreich GmbH (Table S1). The BC was EBC-certified [50] and produced from paper sludge and grain husks at 550 °C (Table S2). The amendments were mixed into the soil at an approximate moisture content of 20% using a 4000 L trommel. Considering the dry weight of the soil and amendments, 2.65%wt VK and 2.41%wt BC were applied. The soil treatments were set up in a randomized two-block design. The experimental set up and treatment handling is further detailed in the Supplementary Material.

Four vegetables were grown on the raised beds: spinach (*Spinacia oleracea* L.), radish (*Raphanus raphanistrum* subsp. *sativus* L.), pak choi (*Brassica rapa* ssp. *chinensis*) and bush beans (*Phaseolus vulgaris* var. *nanus*). Plants were watered as required according to soil water sensors. After harvest, leaves and bulbs were separated, weighted, washed, and dried at 60 °C until a constant weight. The plant samples were ground in a stainless-steel ball-mill prior to acid digestion.

### 2.4. Soil and Plant Analysis

Soil samples were taken at the beginning of the experiment in August 2019 as a composite sample from each treatment; measurements were done in triplicates. In September 2019, soil core samples (100 mL) were taken from each bed in duplicates. In May 2020 soil samples taken from each side of the bed (manured/not manured) using a Puerkhauer soil sampler, combining 7 subsamples of 10 cm depth. Soil samples were air dried, sieved though a 2 mm stainless steel sieve and stored at 4 °C. Leachate collection was done once a year after an extensive irrigation event one day before the sampling. Leachates were filtered, adjusted to 2% HNO_3_ and stored at 4 °C until HM and EDTA were measured. Total metal concentrations as well as their physical parameters were only determined at the beginning of the experiment since they were not expected to change over the course of six months. This was also observed in raised bed experiments using remediated alkaline soil [43,44].

The gravimetric water content was determined by drying 5 g of air-dried soil at 105 °C until a constant weight. All results were converted and reported on a dry weight base. The electrical conductivity was determined in a deionized water extract (1:2.5 *w*/*v* ratio) [51]. Values were converted to the saturated paste equivalent (EC_e_) using the formula from Aboukila and Abdelaty (2017) [52]. Soil pH was determined in deionized water using a 1:2.5 *w*/*v* ratio after shaking for 2 h. The effective cation exchange capacity (CEC_eff_) was determined at pH 7 using a modified version of the method of Burt (2004) [53]. Then, 2.5 g of soil and 40 mL NH_4_OAc were shaken for 1 h until all exchange sites are saturated with NH_4_^+^. The soil was washed free of excess salts with ethanol and NH_4_^+^ adsorbed to the soil matrix extracted using a 0.5 mol L^−1^ K_2_SO_4_ solution, filtered and NH_4_^+^ determined colorimetrically. Inorganic carbon was determined by Scheibler method [54] using 2 g of ground soil. Total carbon and nitrogen (N_tot_) contents were measured by Dumas combustion elemental analysis (Flash 2000, Thermo Scientific, Waltham, MA, USA) [55]. The organic carbon (SOC) content was calculated by subtracting inorganic from total carbon.

Metals extracted using 1 M NH_4_NO_3_ (*w*/*v* ratio 1:2.5) [56] will be referred to as the exchangeable fraction due to its good approximation [57]. Micronutrients, potassium and phosphorous were extracted using Mehlich-3 solution [58]. HMs extracted with Mehlich 3 will be referred to as potentially plant available metals [59]. Total metal concentrations in soil were measured after aqua regia digestion. Then, 0.5 g of air dried soil was digested in a heating block at 135 °C for 3 h using concentrated acid (1.5 mL HNO_3_ 65%, 4.5 mL HCl 37%) [60]. Total metal concentration in plants was measured after acid digestion (5 mL HNO_3_ 65 %, 1 mL H_2_O_2_ 30%) [61]; 0.2 g of over dried sample was digested at 145 °C for 2.5 h.

Total and NH_4_NO_3_ extractable Pb, and for Cd extracted with the Mehlich-3 extract, were measured by graphite furnace–atomic absorption spectroscopy (GF–AAS) (HGA 900 coupled with AAnalyst 400, Perkin Elmer, Waltham, MA, USA). Total and NH_4_NO_3_ extractable Zn was measured using Flame-AAS (AAnalyst 400, Perkin Elmer, Waltham, MA, USA). All other Mehlich 3 extracts were measured using inductively coupled plasma–optical emission spectrometry (ICP–OES) (Optima 8300, Perkin Elmer). Blanks, quality control standards and certified reference materials (Plants: INCT-OBTL-5, Soil: ISE 885) were included in each measurement sequence. The recovery of all target ions ranged between 85–110 % for the reference materials. Detailed measurement conditions and detection limits are listed in the Supplementary Material (Tables S3 and S4). Water-extractable EDTA in soil (1:2 *w*/*v* ratio, shaking time 2 h) and EDTA in leachate was measured by an indirect spectrometric method adapted from Wang et al. (2013) [62] and described in detail by Noller et al. (2021) [41]. The detection limit was 2 mg L^−1^. Soil particle size distribution was determined in duplicates using wet-sieving and particle separation using the Köhn pipette method [63]. Soil aggregate stability was characterized using the wet-sieving method described by DIN 19683-16: 2015 [64]. Total porosity and field capacity were measured using a sand bath and following ÖNORM EN 13041:2011 [65].

### 2.5. Statistical Analysis

The Kruskal–Wallis test and Dunn’s multiple comparison post hoc test with the Bonferroni correction were used to compare the parameter variances between the soil treatments on the same sampling date. Differences between the sampling dates of the same treatment, between radish/spinach cultivars and manured/raw treatments were analyzed using the unpaired Welch *t*-test The statistical analysis was carried out using R Studio [66,67].

## 3. Results

### 3.1. Cultivar Selection

The various vegetables, radish, spinach, carrot and lettuce, took up significantly different concentrations of HM (Figures S4–S7). Among those vegetables, only radish showed significant and reliable differences in metal uptake between cultivars (Figure S5). The Topsi cultivar showed significantly higher uptake of Pb, Cd and Zn compared to most of the other species. French Breakfast and Halbrot-Halbweiß cultivars were among the lowest uptake cultivars for all three metals. For the field experiment two cultivars of radish (Butterflay, Resistoflay) and spinach (Topsi, French Breakfast) were selected for the highest and lowest Pb and Cd uptake.

### 3.2. Soil Properties

#### 3.2.1. Basic Properties

Many basic and nutritional chemical properties were significantly affected by soil washing and the amendment of biochar and vermicompost (Table 1). The soil pH and EC increased significantly in the washed treatments. Salts were introduced by the recycled washing solution and the addition of Na–EDTA, which is needed to compensate for EDTA losses of up to 15 % in each washing batch [43]. High salinity (EC) in treatment WA and W was confirmed by a substantial increase in Mehlich-3-extractable Ca, Na, Fe and S (Table 1). The CEC_eff_ was not changed by the washing treatment but increased significantly in treatment WA.

SOC and N_tot_ contents did not change significantly after soil washing but increased after the organic amendment, raising the C/N ratio from 9.5 in treatment C and 10.1 in W to 16.6 in WA. NH_4_–N increased after soil washing in treatment W but decreased again in WA. NO_3_–N decreased in both W and WA treatments. NH_4_–N and NO_3_–N increased in all treatments from 2019 to 2020 even if not always significantly, N_tot_ content increased significantly in C and WA treatments.

P concentrations were reduced by half in the W treatment but were reintroduced through the organic amendments, finally exceeding the original levels. K increased in the W treatment by half and showed another sixfold increment in treatment WA [68]. P and K significantly decreased from 2019 to 2020 in the W and WA treatments, while no change was observed in treatment C. Na measurements in 2020 failed due to measurement problems.

Texture analysis classified the treatment C as a loam based on the FAO/WRB guidelines [69]. All physical soil parameters investigated were strongly affected by washing and amendments (Table 2). Washing increased the relative clay content, shifting the soil texture towards silt loam in the W treatment. Course material was reintroduced with the compost amendment, clearly classifying the A treatment as loam. Field capacity decreased after soil washing as did bulk density and pore space, while the aggregate stability increased.

#### 3.2.2. Heavy Metal Behavior

The initial total metal concentrations were 795, 4.47 and 484 mg kg^−1^ Pb Cd and Zn, respectively, in contaminated treatment C (Table 3). After soil washing, concentrations significantly decreased to 189 mg kg^−1^ for Pb, 2.36 mg kg^−1^ for Cd and 410 mg kg^−1^ for Zn. Furthermore, extractable fractions were significantly lowered in the washed soils compared to the soil C. Comparing concentrations from treatment C to W, the reduction of Pb, Cd and Zn was 76, 47 and 15%, respectively, in the total fraction. In the NH_4_NO_3_ extract, the reduction was 61, 63 and 97%; in the Mehlich 3 extract it was 84, 68 and 81 %. The addition of amendments further reduced the Pb concentration in the NH_4_NO_3_ extractable fraction significantly. On the second sampling date, NH_4_NO_3_-extractable Pb, Cd and Zn increased significantly in the C treatment and almost doubled in concentration, while in treatment WA and W, only Pb and Zn were significantly reduced.

**Figure 2:**
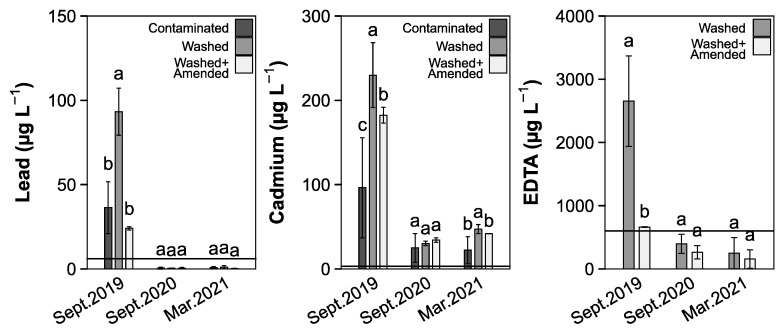
Heavy metal concentration measured in the soil leachate (mean ± SD, *n* = 4). Different lower-case letters indicate differences between the soil treatments of one sampling point (Dunn test, *p* < 0.05).

Zn concentrations in the leachate were mostly below the limit of detection (LOD) throughout the experiment. Pb and Cd exhibited a peak at the beginning of the field experiment in all soil treatments (Figure 2). Initially, Pb leachate concentrations were 37 µg L^−1^, 93 µg L^−1^ and 23 µg L^−1^ in treatments C, W and WA, respectively. Cadmium leachate concentrations were 104 µg L^−1^, 230 µg L^−1^ and 177 µg L^−1^ for C, W and WA, respectively. After 12 months, the concentrations found in the leachate had decreased significantly to 0.35 µg L^−1^ for Pb and 30 µg L^−1^ for Cd, both indifferent between the three treatments. After 19 months, no significant change was recorded. Water-extractable EDTA was 9 mg L^−1^ in treatment W and 2.5 mg L^−1^ in treatment WA and both were significantly reduced after 12 months to 2.3 and 0.4 mg L^−1^. No change in concentration was reported after 19 months.

**Figure 3:**
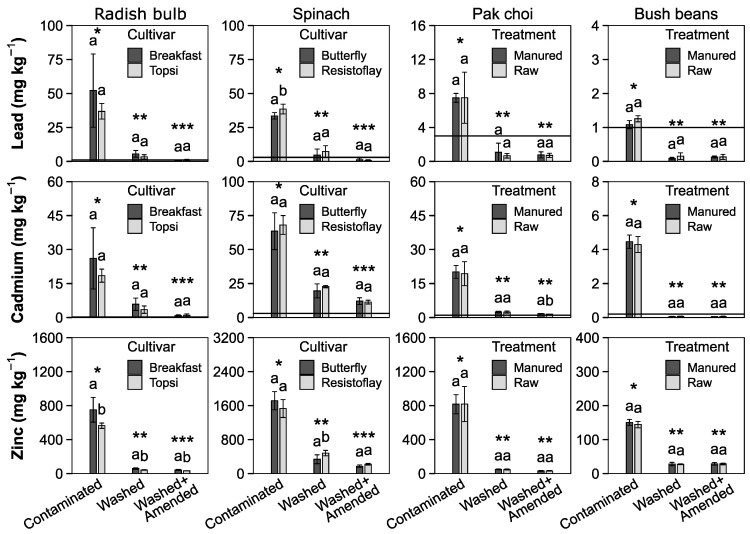
Uptake of heavy metals into eatable parts of different vegetables (mean ± SD, *n* = 4). The dashed line indicates EU guide values adapted for dry weight, assuming 90% water content. Different numbers of asterisks represent significant differences among soil treatments (Welch test, *p* < 0.05). Different lower-case letters indicate significant differences between cultivars and the green manuring in the same soil treatment (Dunn test, *p* < 0.05). Different number of asterisks indicates a significant difference between die soil treatments (Dunn test, *p* < 0.05).

Soil washing significantly reduced the HM uptake in all vegetables (Figure 3). Spinach showed the highest HM uptake compared to the other plants. Bush beans showed the lowest uptake of heavy metals into their fruiting bodies. HM uptake was already low in treatment C and was further reduced by soil washing with no differences found between the W and WA treatments. Generally, the reduction for HM from treatment C to W was over 80% except for Cd in bush beans (67%). The increased NH_4_NO_3_-extractable Pb in the green manured treatment did not result in a significantly higher plant Pb uptake.

### 3.3. Plant Yield

**Figure 4:**
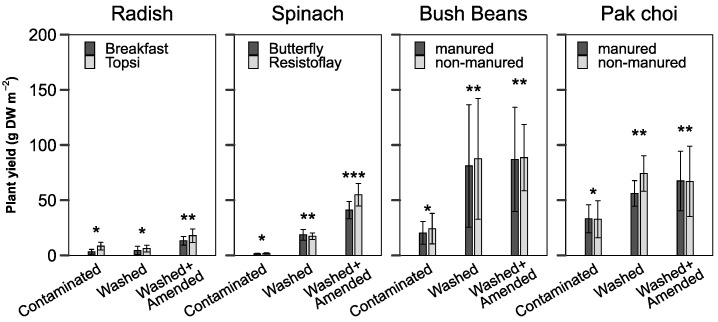
Yields of vegetables grown on contaminated and washed soils (mean ± SD, *n* = 4). For spinach and radish, two cultivars were compared. For pak choi and bush beans, two additional soil treatments were tested. Different number of asterisks indicates a significant difference between the soil treatments (Dunn test, *p* < 0.05).

Plant growth was assessed as dry-harvested biomass (Figure 4). Yields for radish did not change for the W treatment but increased 2.7-fold for the WA treatment. Spinach grew dwarfish on the C treatment and showed strong stress symptoms while yields increased 11-fold on W and 30-fold on the WA soil. Yields for bush beans and pak choi did not significantly vary between both washed treatments but increased four and two-fold, compared to treatment C, respectively.

## 4. Discussion

### 4.1. Impact of Soil Washing on the Soil Fertility

The invasive character of conventional soil-washing technologies alters various physical soil properties. A major difference of the ReSoil^®^ technology when compared to conventional soil washing procedures is the preservation of all soil texture fractions which are important to keep fundamental soil physical properties intact. However, sieving, slurrying and compaction during the dewatering process impacted the soil aggregation and initially led to the complete loss in soil structure. Substantial concentrations in exchangeable Na, K and Mg (Table 1) promoted clay dispersion, adding to the structural deterioration [70,71,72]. Treatment W also showed a significant increase in fine fraction (<2 µm) which is attributed to the mechanical forces and partial loss of sand (>63 µm) during the procedure and has also been observed in other washing technologies [73]. Soil consolidation during the filter pressing and subsequent crumbling of the soil filter cakes resulted in the formation of artificial soil aggregates and clods of high stability (Table 2). This was also facilitated by the introduction of CaSO_4_ (gypsum) during the washing procedure, enhancing clay flocculation and the formation of Ca bonds with clays and organic matter particles [74,75]. The formation of Fe–oxyhydroxides from the ZVI amendment possibly further added to this stabilization as they are important binding agents for soil aggregates [76,77]. High loads of ZVI could lead to negative effects on soil structure due to increased cementation by solidifying larger soil clods [78]. Furthermore, ZVI might have triggered changes in soil bulk density and the water storage capacity. Inferring from the significant decrease in the plant available water content, a significant amount of micropores must have been lost during the washing process. Soil consolidation in the washed treatment W led to the increase in soil bulk density and caused the reduced porosity and field capacity compared to the original soil (Table 2). In treatment WA, a similar reduction in pore space was observed despite a reduction in bulk density, which was attributable to the lower density of the organic amendment [46]. The general loss of porosity in W was also observed by Gluhar et al. (2021) [45]. The pore structure was dominated by macropores, required for the additional irrigation of W and WA treatments to maintain similar plant growth conditions in all treatments.

Besides physical characteristics, soil washing significantly altered basic chemical soil properties. A critical change was caused by the introduction of CaSO_4_, mentioned before, which significantly raised the soil pH by one unit (Table 1). Consequently, the availability of nutrients as well as plant-available HM was altered due to an increase in the retention capacity of the soil [79]. Contrary to this, the introduction of Ca, Fe, Mg, K and Na significantly increased the EC_e_ (Table 1) which can affect the sorption behavior of HMs and increase their plant availability [80].

The organic matter is another important factor in the sorption behavior and nutrient transformation in soil and was also affected by the remediation treatment. EDTA soil washing can result in a partial dissolution of SOC [81] and therefore degrade soil fertility. Jez et al. (2021) [82] found qualitative changes in the SOC composition in EDTA washed soil, and Gluhar et al. (2020) and (2021) [36,43] reported a significant reduction in SOC and measured high dissolved organic matter concentrations in the washing solution. This experiment did not show any significant change in SOC content after EDTA soil washing (Table 1) which seems to align with other studies indicating no change in SOC after EDTA washing [28,83,84]. However, those studies were set up as pot experiments, where the leaching of significant amounts of SOC are unlikely. We cannot exclude a qualitative change in the SOC composition. SOC dissolution and N mineralization is suggested by the significant increase in NH_4_–N concentration at the first sampling date. A decrease in NO_3_–N content in the W treatment might be explained by the extraction of mobile NO_3_–N during the washing procedure (Table 1).

We did not find a negative effect of EDTA soil washing on mineralization and nitrification over the study period. After nine months, the NO_3_–N levels in W increased by +39.1 mg kg^−1^ compared to +27.1 mg kg^−1^ in the C treatment (Table 1) which was most likely a result of increased nitrification at the higher pH following soil washing, which shifted the soil pH closer to the nitrification optimum [85]. Increasing NH_4_–N concentrations were found over all treatments and suggested an increased mineralization of SOC after homogenizing the soil, similar to effects observed after tillage [86]. In the W treatment NH_4_–N only showed a slight increase possibly due to the lower substrate availability and faster nitrification, while lower nitrification in the C treatment led to an accumulation of NH_4_–N (Table 1). We conclude that the washed soils improved the conditions for microorganisms especially for nitrifiers, mostly due to the pH shift, but possibly also in part due to the decreased toxicity. This is a contrast to most studies reporting the inhibiting effects of EDTA and soil washing on microbial activity [87,88] and changes in community composition [38,89].

Other essential nutrients showed significant changes in the washed treatments. While P concentrations in the C treatment showed very high levels according to general guidelines for plant nutrition [68], they were reduced to the lower optimum range after washing (Table 1). K concentrations were found in the very low range for treatment C and increased slightly after washing. After the organic amendment, K values were found at high levels. The decrease in P after soil washing and the simultaneous increase in K could either be explained by an higher affinity of EDTA towards P, leading to a higher extraction, or the high Ca concentrations in the washed treatments combined with an increased pH, which could promote the precipitation of Ca phosphates [90]. The significant increase in K can only be explained by an external input through the ion-rich washing solution. Gluhar et al. (2021) [43] showed the same trend found in our study but reported increasing levels of both nutrients in an earlier experiment [36]. A increase in available P and decrease in exchangeable K has been reported by many studies [73,91,92], explained by the reduction of P fixation by EDTA. Since no recycling of the washing solution took place in these studies, K was not enriched. While loss in nutrients through soil washing should be avoided, it is highly dependent on the specific washing procedure. Over the study period, available P and K concentrations decreased with the removal by plants in the high yield treatments W and WA (Figure 1). The low biomass production in treatment C (Figure 4) was not sufficient to significantly change the concentration of available nutrients (Table 1). In earlier studies, Mn deficiency induced by EDTA soil washing was stated to be a possible threshold for plant production [15,45], but this was not supported by our findings. Sufficient levels of Mehlich 3-extractable Mn were reported in treatment C and even increased significantly in the washed treatments (Table 1).

### 4.2. Impact of Soil Washing on Plant Biomass Production

The soil remediation was not only aiming to reduce HM to acceptable levels, but also to restore soil conditions suitable for vegetable production. Besides the overall positive changes in nutrient availability in the remediated soil explored above, the effect of residual amounts of EDTA on the plant availability of HM and the substantial introduction of salts were the two main concerns for a negative impact in plant growth. High Ca, Na and S concentrations and EC_e_ (Table 1) could lead to growth depressions for many vegetables [93]. However, no visible salinity stress symptoms developed when vegetables were grown on the remediated soil.

EDTA was still present in the treatments W and WA in residual concentrations (Table 1). It is regularly used as a fertilizer (e.g., Zn-EDTA) in countries such as the USA or India and at low concentrations no acutely phytotoxicity effects would be expected [38,94]. It may even enhance microbial growth [95]. However, its longevity in soils can lead to a secondary increase in HM availability and significant biotoxicity [87,96]. Grčman et al. (2001) [97] showed that an increase in HM availability after a single dose of 10 mmol EDTA kg^−1^ led to necrosis in pak choi. In our study, no visual toxic indicators were recorded for plants grown on treatments W and WA, especially due to the successful immobilization of EDTA–metal complexes by Fe [29]. Spinach, bush beans and pak choi even significantly increased in yield when grown on the washed soils when compared to plants in treatment C, where high concentrations of HM led to plant necrosis and dwarfism (Figure S3). These results clearly confirmed the positive effect of soil washing on the plant productivity.

The compost amendment provided additional plant available nutrients P, K and N (Table 1) which further increased the yield of spinach and radish plants compared to W (Figure 4). This advantage ceased in the second growing season after all treatments were fertilized, compensating for the differences in nutrient availability. Therefore, pak choi and bush beans did not show any yield differences between treatment W and A.

Not only nutrient availability but also the physical properties of the soils can impact plant development. The properties of the original soil were changed into an artificial soil with a coherent structure. While it seems straight forward to ascribe the increase in plant yield recorded on the washed soils to the drastically decreased metal concentrations in most vegetables (Figure 1), the fundamental alteration of the soil structure could be equally important [98,99]. The big soil clods formed during soil washing predominantly form macro pores that are easy to be accessed by plant roots [100]. The negative effects of water stress due to less contact with soil and fewer middle-sized pores were compensated for by higher irrigation rates. In hindsight, this is probably one of the most important limiting factors for plant growth to be considered in the future, as water stress was commonly reported in soil washing studies [28,43].

The introduction of Phacelia as green manure was deemed to moderate the disadvantageous physical properties of the washed treatment but was also applied to the contaminated soil. This led to a significant increase in the NH_4_NO_3_-extractable Pb in soil in the C treatment, while Cd and Zn showed a trend of increasing concentrations. Gluhar et al. (2021) showed the same increased in metal bioavailability, due to the release of metals accumulated by the buck wheat used as green manure [43]. Mulching of plant parts produced on the washed treatments were within safe limits and could help to restore the soil structure [101].

### 4.3. Behavior of EDTA and Heavy Metals in the Soil System

Remediation of garden soil can only be successful if its functions as a growth medium is guaranteed. However, the safety of the technology depends on the stability of HM, and a transfer into the food cycle though contaminated vegetables or the contamination of surface and groundwater is not acceptable. The mobility of HM is mainly driven by residual EDTA before the introduction of ZVI [29] but it is still a major concern until its complete stabilization by Fe–oxyhydroxides. Other technologies also aim to reduce the potential of this secondary pollution [102,103]. EDTA concentrations found in soil water extracts were low and similar to an earlier pot experiment [41], indicating the success of the ZVI amendment in reducing residual EDTA through the adsorption of negatively charged EDTA–metal complexes onto Fe–oxyhydroxides [104,105]. Immobilizing residual EDTA is essential for the successful reduction of bioavailable Pb particularly due to its high complex formation constant with EDTA (log K = 18.0) compared to Cd and Zn (both log K = 16.5) [106]. The immobilization of EDTA is not as crucial to Cd availability when compared to Pb since it can already occur in very mobile complexes such as CdCl^+^ and Cd(SO_4_)_2_^2−^, with higher plant availability even at a neutral pH [107]. This concept is confirmed by the large Mehlich 3-extractable portion (46% of the total Cd) in the W treatment compared to Pb and Zn (29% and 5% of the total concentration).

To understand the extent to which metals of different soil fractions can be extracted by EDTA washing, Gluhar et al. (2020) conducted a Tessier sequential extraction [108] on treatment C and a soil similar to W; however, the remediation was conducted on lab-scale (14 kg per batch) [36]. It revealed that Zn was largely retained in the residual fraction prior to soil washing and increased to two-thirds after the treatment. Generally, only small amounts of Zn were found in the exchangeable or water soluble soil fraction [109] and is therefore only of little concern even at very high concentrations. The remaining Tessier fractions were almost completely removed with concentrations similar to the NH_4_NO_3_-extractable Zn in treatments W and WA (Table 3).

The biggest portion of Pb was successfully removed from the exchangeable, inorganic and oxide fraction and the removal of half of the organic Pb fraction was high compared to Cd and Zn [36]. NH_4_NO_3_-extractable Pb was reduced 2.5-fold and between the two sampling dates, the availability of Pb and Zn was further reduced significantly in the W treatment. This continuing decrease in available Pb was the result of the typical two-staged adsorption process of metals onto Fe–oxyhydroxides. After a rapid sorption occurs in the first days after the addition of ZVI, reaching a pseudo equilibrium at the mineral–water interface, a much slower sorption process can continue for years [110]. Simultaneously, the advancing ZVI oxidation forms new Fe–oxyhydroxides participating in the sorption of Zn, Pb or EDTA–Pb complexes [111] and even decreased the Mehlich 3-extractable Pb on the second sampling date (Table 2). The organic amendment led to an additional significant decrease in NH_4_NO_3_-extractable Pb (Table 2). This coincided with a significant increased CEC in treatment A (Table 1) which increased the adsorption of Pb onto soil organic matter. Due to their high affinity toward Pb [112] organic matter amendments have successfully been used for remediating polluted soils before [113,114,115] but only mixed results were reported for Cd and Zn [116].

Bioavailable Cd is actively taken up by plants [117] and therefore transfer factors are higher compared to other HM [118]. In the contaminated soil, Cd was mainly present in the exchangeable Tessier fraction [36] and was almost completely removed by the soil washing. However, it is characterized by faster desorption kinetics which resulted in a quicker supply from the solid soil fraction compared to Pb and Zn [112,119]. Cd is also less dependent on soil pH [120] and therefore very mobile. This could have been reinforced by a high salt concentration in the soil solution, which is one of the key factors of controlling Cd sorption [121]. High salt contents increase the competition for sorption sites [80] and might have also led to the desorption of HM ions into the soil solution. Especially Ca and K are effectively competing for sorption sites with Cd [122], which were both increased after soil washing (Table 1). This explains the constant concentration of Cd on the second sampling date compared to the decrease in available Pb and Zn due to advancing adsorption. A similarly high Cd plant uptake despite the increasing ZVI amendments was reported in one of our earlier studies [41]. The significant increase in NH_4_NO_3_ extractable metals in the C treatment on the second sampling date was unexpected but could be related to heterogeneity of the material or the mineralization of SOC induced by the soil mixing, discussed before, and would partly explain the increase in bioavailable metals, as they are released from the organic matter.

To conclude, soil washing and ZVI amendment successfully reduced the metal concentrations in the total as well as in the NH_4_NO_3_ extractable fraction, however, it failed to match the national soil guideline values (Total: Pb 100, Cd 0.5 and Zn 300; NH_4_NO_3_: Pb 0.3, Cd 0.04, Zn 4.0 in mg kg^−1^) for vegetable production [123]. To apply this technology for garden soils, significant improvements have to be made in the washing process or post-treatment. Our data showed that the extraction of total HM was lower compared to earlier studies [36,41]. While this study showed a 2.5-fold reduction of the NH_4_NO_3_-extractable Pb in treatment W, Gluhar et al. (2020) reported a 50-fold reduction [36]. This discrepancy was most likely due to the different scale, since the soils in the previous studies were washed in smaller-scaled batches, where experimental conditions were easier to control. In both studies, a higher reduction in total Cd and NH_4_NO_3_-extractable Pb and Cd fractions was achieved, while the latter also showed a higher reduction in total Pb. The lower extraction effectivity could be due to decreasing concentrations of EDTA in the washing solution over time, or the saturation of the processing water with salts from previous batches. Such an enrichment was found in the Mehlich 3 extract (Table 3) and could lead to higher competition for the EDTA complex formation. Previous studies have also shown that the extraction efficiency of the pilot plant can vary between batches due to technical problems during the washing process. Gluhar et al. (2021) reported a poor separation of washing solution from the soil slurry during the filter pressing as a possible explanation [43]. It was caused by the incomplete filling of the filtration chambers and will be improved in future applications. Compared to other studies applying different EDTA soil washing technologies, the reduction in bioavailable Pb, Cd and Zn was well within common removal efficiencies [32] but results are difficult to compare due to differing process parameters such as the EDTA concentration and liquid–solid ratios used, as well as the specific soil characteristics.

### 4.4. Heavy Metal Uptake into Plants

As stated previously. the uptake of HM by vegetable consumption has to be minimized and to assess the safety of the food products produced on remediated soil, national and international guide values have great importance. Despite exceeding the Austrian state recommended values for total and extractable HM, the reduced values resulted in significantly lower plant uptake for all vegetables (Figure 3). Only in bush beans grown on the washed treatments W and WA did Pb, Cd and Zn meet the EU guidelines set by the European Commission regulations 2021/1317 and 2021/1323. For radish, spinach and pak choi, only the Pb concentrations met the thresholds when grown on treatment WA.

The significant reduction in HM uptake by all vegetables grown on treatment W was well explained by the reduced bioavailability after the soil washing and was indicated by the NH_4_NO_3_-extractable HM (Table 2). The additional significant decrease of Pb and Cd in spinach and radish plants grown on treatment WA was only reflected in the extractable fraction of Pb. The significant reduction of Cd uptake on treatment WA was probably due to a dilution effect [124]. Nutrients introduced by the organic amendment led to a faster growth and lowered the uptake of HM per unit of produced biomass [125,126]. Fertilizing before the second growth period nullified this effect so bush beans and pak choi did not differ in yield nor HM uptake between the treatments W and WA.

The high metal concentrations in spinach plants and lower concentrations in radish bulbs are known from other studies [127]. The continuous stabilization of Pb and Zn, discussed earlier, explained the reduced concentration in the pak choi and bush bean plants, but most importantly, the lowest concentrations were found in the fruit bodies of the bush beans, which do not enrich HM with the xylem stream, but are supplied through the phloem transport, which is a physiological barrier for many HMs [128].

Cultivar selection had a variable impact; some of the radish and spinach cultivars took up higher amounts of HM compared to the mean concentration in the greenhouse setting. However, none of these differences were reproducible when plants were grown in the raised beds (Figure 3). Significant differences in the Cd uptake between the radish cultivars Topsi and French Breakfast were detected in the WA and W treatment but in reverse order compared to the pot experiment (Figure S5). Although significant, the differences were most like due to a dilution effect: While Topsi plants showed a trend for higher yields (Figure 4) and lower metal uptake (Figure 3) in the raised beds, the reverse was found in the pot experiment (Figure S8). This merely showed a possible yield advantage of the French Breakfast cultivar under field conditions. However, pollution-safe cultivars could be an option for future applications and need directed breeding efforts to result in significant exclusion capabilities [129].

### 4.5. EDTA and Heavy Metals in the Soil Leachate

The trace concentrations of EDTA in soil showed its minor influence on the plant uptake of HM. However, it is possible that mobile EDTA–metal complexes were already washed out from the root zone before becoming accessible by plant roots. EDTA is very persistent in soil and its resistance to chemical and biological degradation processes is well documented [130]. The high leachability of EDTA and metal–EDTA complexes into the subsurface or groundwater would occur without stabilizing these compounds though the ZVI amendment [15,38,131]. Accordingly, Gluhar et al. (2021) detected no HM in the soil leachates after ZVI amendments [43]. However, high initial EDTA mobility early into the experiment or desorption form the Fe–oxyhydroxides at a later point could still lead to high metal mobility even beyond the concentration found in the originally contaminated soil.

On the first sampling date, residual EDTA was found in the leachate in W and to a lesser extend in WA, suggesting an incomplete sorption of the EDTA complexes onto the Fe–oxyhydroxides or a lack of sorption sites due to an incomplete aging of the ZVI (Figure 2). In earlier experiments, EDTA concentrations were shown to subside after three weeks [29]. However, our results suggest that the aging time or the available sorption sites on the ZVI are not sufficient to stop the HM washout. In treatment W, this caused Pb and Cd concentrations to be above those found for the contaminated treatment C. Treatment WA only showed elevated Cd concentrations while Pb concentrations were successfully reduced compared to the control. The organic amendment adsorbed additional EDTA–metal complexes, again showing that Pb availability is driven by EDTA activity, while Cd was still highly available even if not complexed by EDTA. Over time the concentrations of Pb and Cd were indifferent to the original soil due to complete complexation or washout of the mobile metals. A longer aging period or additional amendments will be needed to reduce the HM leaching, since EDTA and Pb concentrations did only fall within WHO drinking water guide values (EDTA: 600 µg L^−1^, Pb: 10 µg L^−1^, Cd: 3 µg L^−1^ [132]) after an initial spike, but Cd concentrations did not match acceptable levels.

The scale up of the treatment technologies and the experimental design revealed that even if principles are well understood, changes in scale can lead to strong variations in the results of plant yield, HM uptake, as well as soil chemical and physical properties. This makes extrapolations and recommendations based on small-scaled experiments extremely difficult. It is generally observed that in small-scale set ups, the liquid–solid ratio is very wide, ranging from 1 to 40, and EDTA levels are usually not calculated per gram of soil [133,134,135]. This leads to very high concentrations of EDTA in the washing solutions, resulting in high extractions rates; this has already been studied in detail [33,34]. Furthermore, no results on residual or leachable EDTA concentrations are included for most publications on soil-washing technologies; this makes it difficult to compare the success of these studies, since reducing residual EDTA mobility has not been widely considered yet.

## 5. Conclusions

This study demonstrated the effect of EDTA soil washing on important soil properties, plant production and risks, with special reference to groundwater contamination. The findings report that bush beans produced in remediated soil could meet EU guideline values for the safe production of vegetables after vermicompost- and biochar-amended treatments, however, soil extractable metal fractions did not meet national recommended levels. Improvement of the washing procedure and a longer aging period after the treatment are strongly recommended to prevent initial peaks in EDTA and metal leaching and further reduce concentrations below the national recommended values. The biggest problem was a high availability of Cd in all soil fractions and continuous leaching, whereas Pb and Zn were shown to be stable over a longer period. Reduced heavy metal availability and improved soil fertility both led to an increased vegetable yield. The amendment of compost further increased vegetable yields and fertilization is recommended after EDTA soil washing. Selection of commercially available vegetable cultivars had no significant impact on the metal uptake. The soil structure was fundamentally changed after the washing treatment. The dissolution of soil aggregates led to the decrease in fine and middle pores, decreasing the plant-available water content of the remediated soil. Organic amendments could not rehabilitate the soil structure during the two experimental years. The biggest challenge for future applications will be the restoration of the soil water storage capacity.

## Figures and Tables

**Table 1:** Chemical characteristics of the soil treatments at the start of the experiment and after 7 months (mean ± SD, *n* = 4). Lower case letters indicate significant differences between groups of one sampling event. Asterisks indicate significant differences between 2019 and 2020 of one treatment.

	Contaminated	Washed	Washed + Amended	Contaminated	Washed	Washed + Amended
		2019			2020	
pH (water)	5.86 ± 0.16 c	7.14 ± 0.01 b	7.34 ± 0.01 a	5.46 ± 0.04 c	7.13 ± 0.03 b	7.20 ± 0.02 a *
EC_e_ mS cm^−1^	2.25 ± 0.23 b	8.03 ± 0.05 a	7.75 ± 1.12 a	2.90 ± 0.83 c	11.6 ± 0.68 b *	12.8 ± 0.87 a *
CEC_eff_ (cmol_c_ kg^−1^)	10.4 ± 0.17 a	10.7 ± 0.07 a	11.8 ± 0.13 b	10.3 ± 0.09 a	10.4 ± 0.26 a	11.5 ± 0.10 b
CN ratio	9.5 c	10.1 c	16.6 a	8.8 b	8.5 c	14.3 a
SOC (%)	2.86 ± 0.11 b	2.93 ± 0.27 b	5.31 ± 0.58 a	3.18 ± 0.14 b	2.76 ± 0.07 c	5.44 ± 0.7 a
N_tot_ (%)	0.30 ± 0.01 a	0.29 ± 0.03 a	0.32 ± 0.03 a	0.36 ± 0.01 a *	0.29 ± 0.02 b	0.38 ± 0.01 a
P (mg kg^−1^)	50.8 ± 1.62 b	27.4 ± 0.58 c	58.0 ± 1.76 a	50.6 ± 1.39 a	18.5 ± 2.90 c *	37.1 ± 4.19 b *
K (mg kg^−1^)	75.9 ± 3.73 c	110 ± 2.50 b	511 ± 23.02 a	73.4 ± 5.36 c	89.1 ± 4.66 b *	456 ± 46.8 a
NH_4_-N (mg kg^−1^)	8.64 ± 1.57 a	10.7 ± 0.32 a	7.83 ± 1.38 a	18.3 ± 3.27 a *	11.6 ± 0.33 b *	9.88 ± 1.56 c
NO_3_-N (mg kg^−1^)	75.9 ± 30.8 a	52.9 ± 6.7 a	60.7 ± 11.1 a	103 ± 45 a	92.0 ± 22.3 a *	154 ± 42.0 a *
EDTA (mg kg^−1^)	n.d.	42.2 ± 1.57 a	37.4 ± 5.17 a	n.d.	15.7 ± 3.56 a *	17.6 ± 4.61 a *
Ca (mg kg^−1^)	883 ± 69.8 b	3360 ± 92.2 a	3624 ± 201 a	713 ± 77.0 c	3380 ± 82.2 b	3950 ± 175 a
Fe (mg kg^−1^)	190 ± 14.1 b	583 ± 11.2 a	567 ± 10.9 a	113 ± 2.94 b *	384 ± 14.1 a *	377 ± 5.28 a *
Mg (mg kg^−1^)	118 ± 4.75 b	72.8 ± 2.31 c	146 ± 6.77 a	149 ± 23.6 b	133 ± 6.52 b *	168 ± 16.3 a
Mn (mg kg^−1^)	32.6 ± 1.34 c	78.9 ± 1.27 a	68.2 ± 2.66 b	33.9 ± 1.17 a	30.7 ± 2.76 b *	36.3 ± 2.22 a *
Na (mg kg^−1^)	62.2 ± 8.22 a	656 ± 14.1 b	552 ± 33.1 c	-	-	-
S (mg kg^−1^)	154 ± 25.9 c	1627 ± 44.1 a	1440 ± 86.3 b	24.9 ± 2.09 b *	1100 ± 52 a *	1120 ± 76.7 a *

n.d.: not detected.

**Table 2:** Physical properties of the soil treatments at the start of the experiment (mean ± SD, *n* = 4). Lower case letters indicate significant differences between groups (Dunn test *p* < 0.05). Soil texture was determined in duplicates.

	Contaminated	Washed	Washed + Amended
Sand (%)	38.2	32.8	40.6
Silt (%)	47.2	49.9	43.4
Clay (%)	14.6	17.3	16.0
SAS ^1^ (%)	79.5 ± 0.83 a	84.4 ± 0.4 b	83.3 ± 0.53 b
Bulk density (g cm^−3^)	1.11 ± 0.02 b	1.17 ± 0.03 c	1.06 ± 0.03 a
Total porosity (%)	55.2 ± 2.30 b	46.2 ± 1.62 a	49.9 ± 4.30 a
Field capacity (%)	38.5 ± 9.8 b	32.6 ± 3.1 a	31.5 ± 3.2 a

^1^ SAS: Soil Aggregate Stability.

**Table 3:** Total, NH_4_NO_3_ extractable, and potentially plant available (Mehlich-3) metal pools (mean ± SD, *n* = 4). Lower case letters indicate significant differences between groups of one sampling event. Asterisks indicate significant differences between 2019 and 2020 of one treatment.

	Contaminated	Washed	Washed + Amended	Contaminated	Washed	Washed + Amended
Total metals (mg kg^−1^)		2019			2020	
Pb	795 ± 22.0 a	189 ± 13.6 b	201 ± 3.0 b	-	-	-
Cd	4.47 ± 0.24 a	2.36 ± 0.09 b	2.14 ± 0.17 b	-	-	-
Zn	484 ± 10.5 a	410 ± 7.05 b	373 ± 9.67 c	-	-	-
NH_4_NO_3_ (mg kg^−1^)						
Pb	3.54 ± 0.15 a	1.37 ± 0.02 b	0.90 ± 0.07 c	8.27 ± 1.00 a *	0.87 ± 0.05 b *	0.48 ± 0.11 c *
Cd	0.60 ± 0.0 a	0.22 ± 0.03 b	0.19 ± 0.03 b	1.03 ± 0.063 a *	0.27 ± 0.014 b	0.24 ± 0.021 b
Zn	25.3 ± 0.72 a	0.68 ± 0.03 c	0.96 ± 0.02 b	45.8 ± 3.64 a *	0.28 ± 0.037 b *	0.23 ± 0.024 b *
Mehlich3 (mg kg^−1^)						
Pb	351 ± 10.5 a	55.3 ± 1.80 b	53.9 ± 1.61 b	321 ± 5.52 a	47.2 ± 3.64 b *	46.19 ± 2.88 b *
Cd	3.42 ± 0.03 a	1.09 ± 0.05 b	1.05 ± 0.03 b	3.22 ± 0.21 a	0.88 ± 0.03 b *	0.87 ± 0.05 b
Zn	112 ± 2.37 a	21.6 ± 0.37 b	20.9 ± 0.66 b	103 ± 2.16 a *	21.0 ± 0.65 b	19.9 ± 0.5 c

## Data Availability

The data presented in this study are openly available in FigShare at https://doi.org/10.6084/m9.figshare.20449251.v1, accessed on 27 October 2022.

## Supplementary Materials

The following supporting information can be downloaded at: https://www.mdpi.com/article/10.3390/toxics10110652/s1. Figure S1: Construction of the raised bed experiment, Figure S2: Homogenization of soil and application of amendments, Figure S3: Toxicity symptoms and growth reduction of vegetables, Figure S4: Spinach cultivars metal uptake, Figure S5: Radish cultivars metal uptake, Figure S6: Carrot cultivars metal uptake, Figure S7: Lettuce cultivars metal uptake, Figure S8: Yields of radish cultivars, Table S1: Chemical characteristics of the vermicompost amendment, Table S2: Chemical characteristics of the biochar amendment, Table S3: Measurement conditions for the atomic absorption spectroscopy, Table S4: Limit of detection for all elements measured using AAS-GF/F and ICP-OES.
