# Supplementary material for: Remediating Garden Soils: EDTA-Soil Washing and Safe Vegetable Production in Raised Bed Gardens
Source: Toxics. 2022 Oct 29;10(11):652. doi: 10.3390/toxics10110652 (PMC9696853; doi:10.3390/toxics10110652)
Supplement: Supplementary file 1 [file toxics-10-00652-s001.zip › toxics-2000489-supplementary.pdf]

Remediating Garden Soils: EDTA-Soil Washing and Safe Vegetable Production in Raised Bed Gardens

Christoph Noller*, Wolfgang Friesl-Hanl, Rebecca Hood-Nowotny, Andrea Watzinger

Department of Forest- and Soil Sciences, Institute of Soil Research, University of Natural Resources & Life Science (BOKU), 1180 Vienna, Austria

***Corresponding author**

christoph.noller@boku.ac.at

University of Natural Resources and Life Sciences

Institute of Soil Research

Konrad-Lorenz-Strasse 24

1180 Vienna, Austria

	Legend	Page
Figure S1	Construction of the raised bed experiment	2
Figure S2	Homogenization of soil and application of amendments	2
Figure S3	Toxicity symptoms and growth reduction of vegetables	3
Figure S4	Spinach cultivars metal uptake	3
Figure S5	Radish cultivars metal uptake	4
Figure S6	Carrot cultivars metal uptake	4
Figure S7	Lettuce cultivars metal uptake	5
Figure S8	Yields of radish cultivars	5
Table S1	Chemical characteristics of the vermicompost amendment	6
Table S2	Chemical characteristics of the biochar amendment	6
Table S3	Measurement conditions for the atomic absorption spectroscopy	6
Table S4	Limit of detection for all elements measured using AAS-GF/F and ICP-OES	6

Supplementary

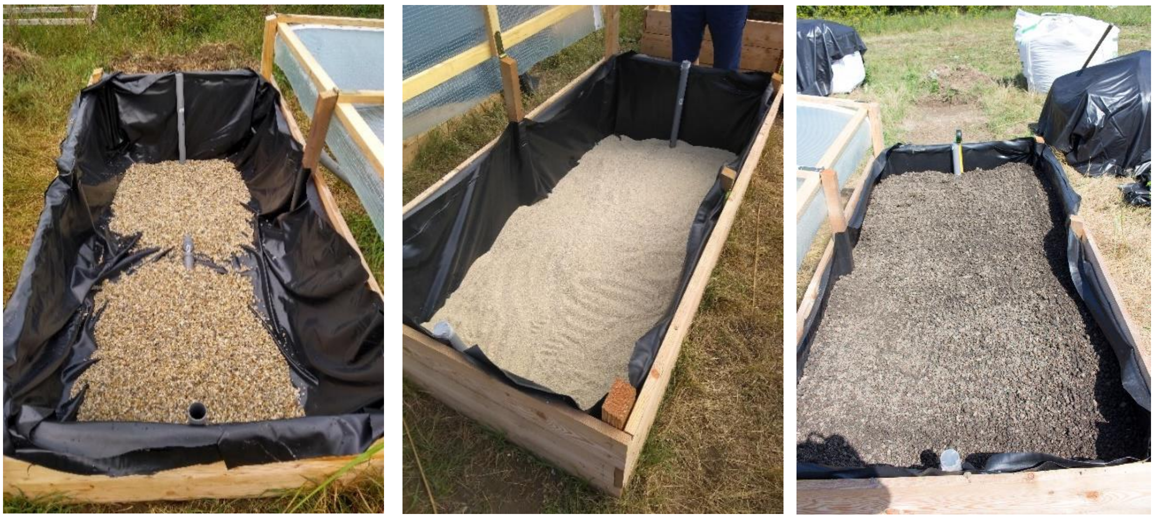

Figure S1: Filling of the raised beds: gravel layer (left), sand layer (middle), soil layer (right).

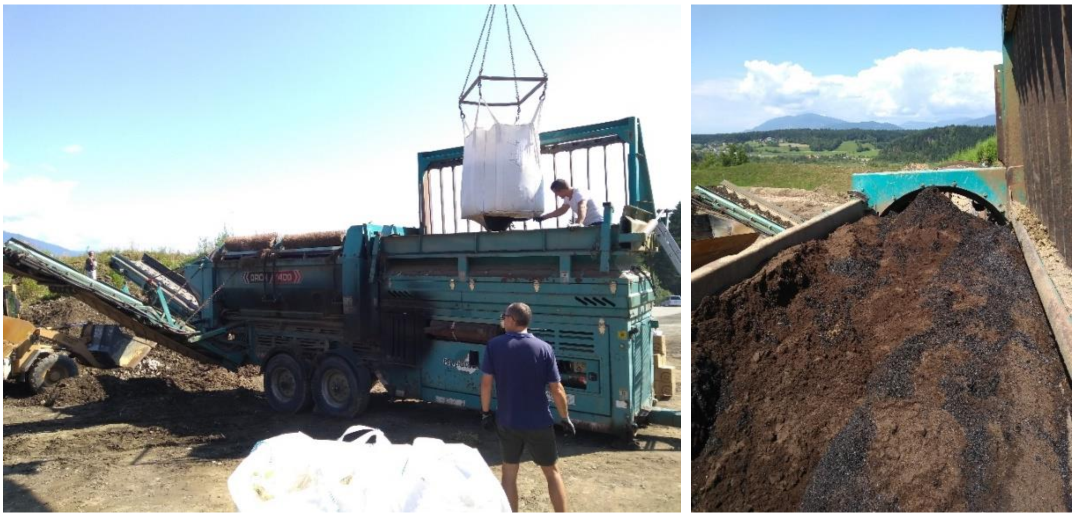

Figure S2: Trommel sieve for mixing the soils (left) and addition of the organic amendments (right).

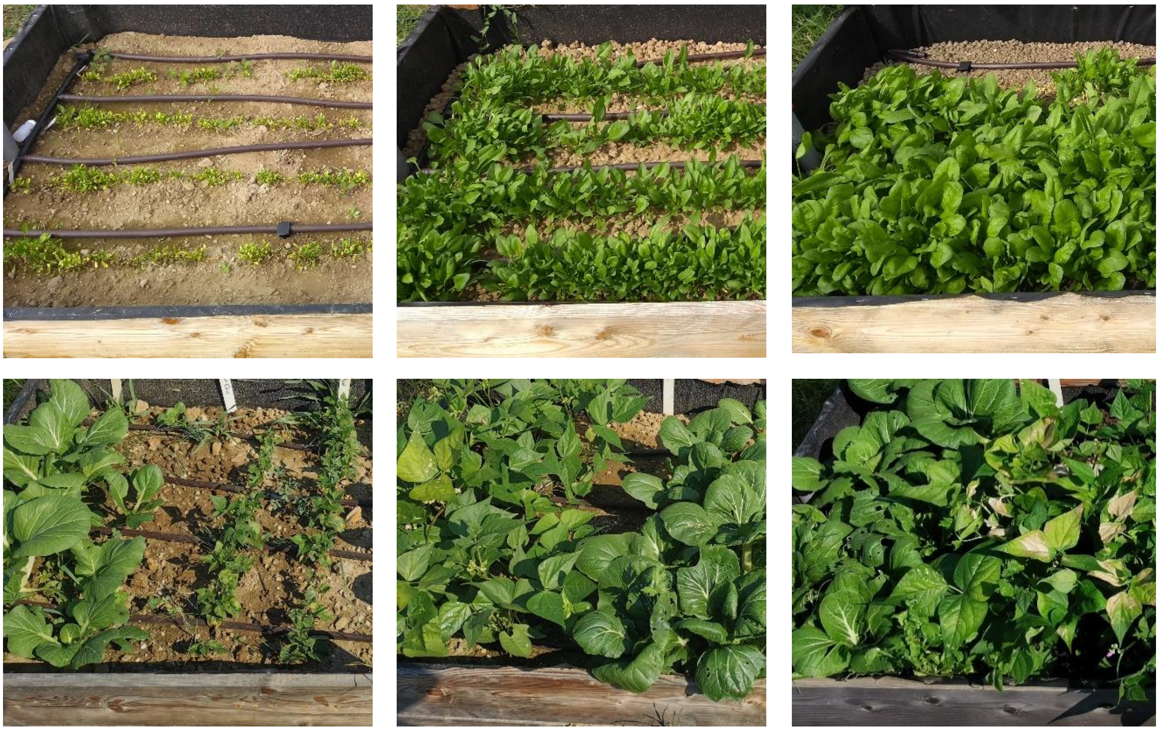

Figure S3: Spinach (upper row), bush beans and pak choi (lower row) before harvest. Plants in the contaminated soil (leaft column) showing toxicity symptoms and dwarfish growth, compared to the washed (middle row) and amended (right row) treatment.

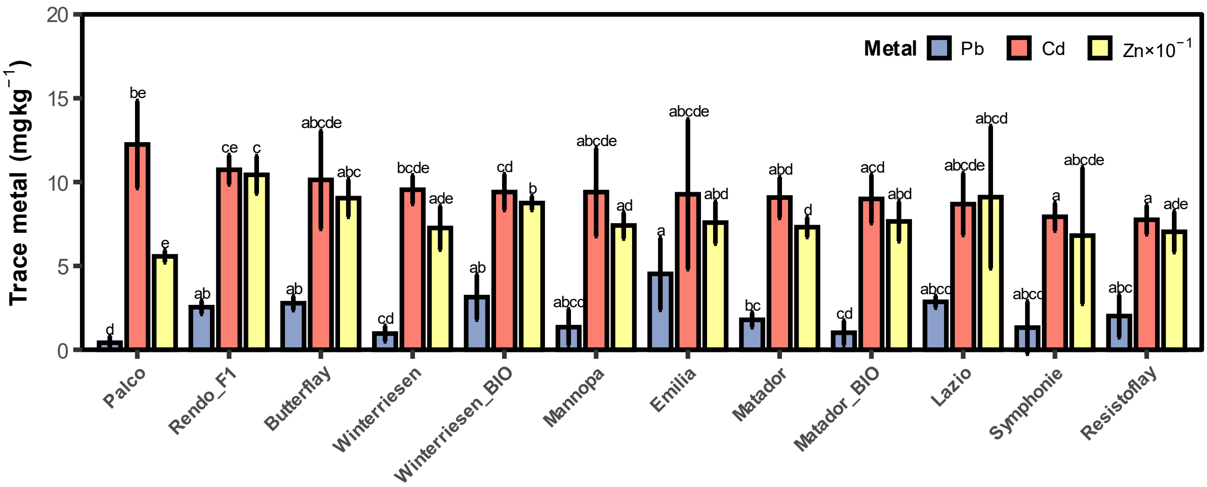

Figure S4: Uptake of heavy metals into spinach cultivars (mean ± SD, n = 4). Different lower-case letters indicate significant differences between cultivars.

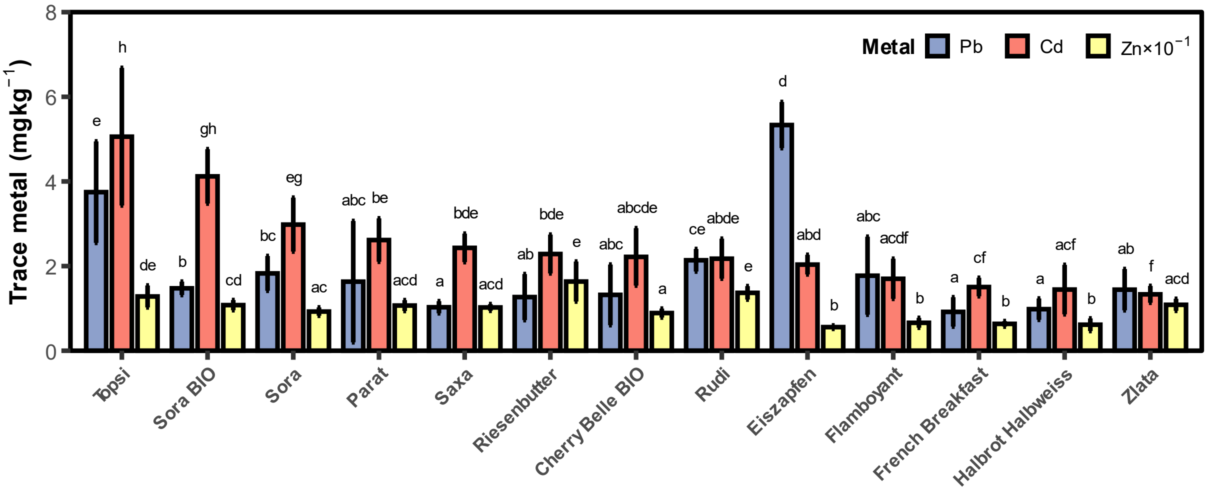

Figure S5: Uptake of heavy metals into radish cultivars (mean ± SD, n = 4). Different lower-case letters indicate significant differences between cultivars.

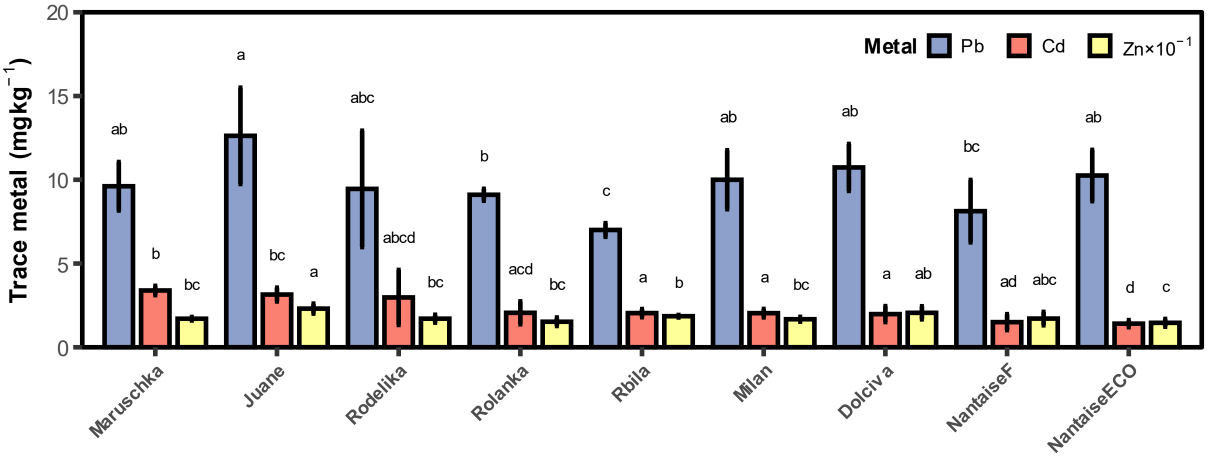

Figure S6: Uptake of heavy metals into carrot cultivars (mean ± SD, n = 4). Different lower-case letters indicate significant differences between cultivars.

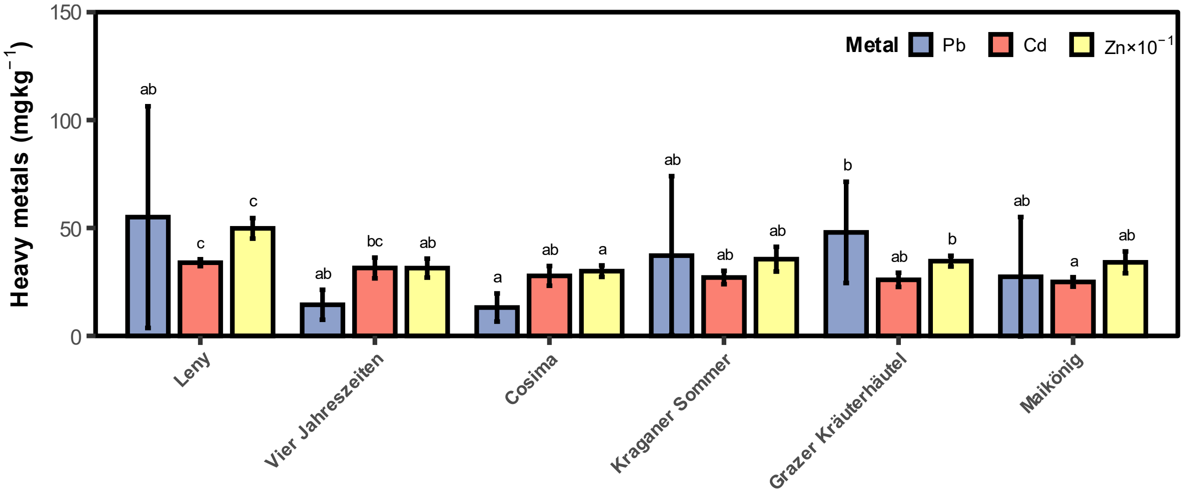

Figure S7: Uptake of heavy metals into lettuce cultivars (mean ± SD, n = 4). Different lower-case letters indicate significant differences between cultivars.

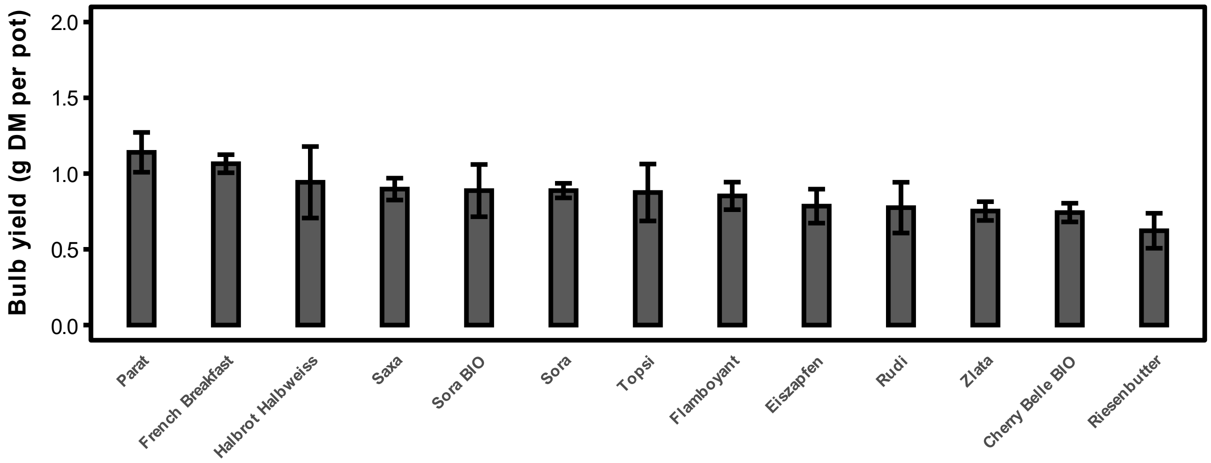

Figure S8: Yields of different radish cultivars (mean ± SD, n = 4).

Table S1: Chemical characteristics of the vermicompost amendment analyzed by EUROFINS Umweltanalytik GmbH.

pH	C_{org}	N_{tot}	P_2O_5	K_2O	MgO	CaO	NO_3	NH_4	Pb	Cd	Zn
	%	%	g kg^{-1}	g kg^{-1}	g kg^{-1}	g kg^{-1}	mg l^{-1}	mg l^{-1}	mg kg^{-1}	mg kg^{-1}	mg kg^{-1}
7.7	12.96	1.08	5.7	14.5	14.1	61.7	5.99	121	15	0.56	98

Table S2: Physicochemical characteristics of the biochar amendment provided by the production company (Sonnenerde GmbH). Nutrients in biochar are mainly present in forms unavailable to plants.

Organic C	Total N	H/C_{org} ratio	P_2O5	K_2O	MgO	Surface area
g kg^{-1}	g kg^{-1}		g kg^{-1}	g kg^{-1}	g kg^{-1}	m^2 g^{-1}
632	9.9	0.17	56	50	31	297

Table S3: Measurement conditions for the atomic absorption spectroscopy.

Element	Utility	Lamp	Background Correction	Matrix Modifier	Wavelength	Carbonization	Atomization	LOD
					nm	°C	°C	mg L^{-1}
Pb	GF	HCL	D2	$NH_4H_2PO_4$	283.3	900	1900	0.5
Cd	GF	HCL	D2	$Pd\text{-}Mg(NO_3)_2$	228.8	650	1600	0.06
Zn	F	HCL	D2	-	213.9	-	2150-2300	2.2

Table S4: Limit of detection for all elements measured using AAS-GF/F and ICP-OES.

Element	Technique	Wavelength (nm)	LOD (mg L^{-1})
Pb	AAS-GF	283.3	0.500
Cd	AAS-GF	228.8	0.060
Zn	AAS-F	213.9	2.200
Ca	ICP-OES	317.933	0.126
Fe	ICP-OES	238.204	0.043
K	ICP-OES	766.490	0.341
Mg	ICP-OES	285.213	0.030
Mn	ICP-OES	257.610	0.005
P	ICP-OES	589.592	0.382
Pb	ICP-OES	214.914	0.178
Si	ICP-OES	220.353	0.005
Zn	ICP-OES	180.669	0.144
